# Postnatal Proteasome Inhibition Induces Neurodegeneration and Cognitive Deficiencies in Adult Mice: A New Model of Neurodevelopment Syndrome

**DOI:** 10.1371/journal.pone.0028927

**Published:** 2011-12-12

**Authors:** Rocío Romero-Granados, Ángela Fontán-Lozano, Francisco Javier Aguilar-Montilla, Ángel Manuel Carrión

**Affiliations:** Departamento de Fisiología, Anatomía y Biología Celular, Universidad Pablo de Olavide, Sevilla, Spain; Ohio State University, United States of America

## Abstract

Defects in the ubiquitin-proteasome system have been related to aging and the development of neurodegenerative disease, although the effects of deficient proteasome activity during early postnatal development are poorly understood. Accordingly, we have assessed how proteasome dysfunction during early postnatal development, induced by administering proteasome inhibitors daily during the first 10 days of life, affects the behaviour of adult mice. We found that this regime of exposure to the proteasome inhibitors MG132 or lactacystin did not produce significant behavioural or morphological changes in the first 15 days of life. However, towards the end of the treatment with proteasome inhibitors, there was a loss of mitochondrial markers and activity, and an increase in DNA oxidation. On reaching adulthood, the memory of mice that were injected with proteasome inhibitors postnatally was impaired in hippocampal and amygdala-dependent tasks, and they suffered motor dysfunction and imbalance. These behavioural deficiencies were correlated with neuronal loss in the hippocampus, amygdala and brainstem, and with diminished adult neurogenesis. Accordingly, impairing proteasome activity at early postnatal ages appears to cause morphological and behavioural alterations in adult mice that resemble those associated with certain neurodegenerative diseases and/or syndromes of mental retardation.

## Introduction

Postnatal brain development is a critical period during which synaptic connections are formed and refined. In the rodent hippocampus, many of these connections reach maturity by the end of the second postnatal week, following an identifiable developmental progression that appears to be common to a wide variety of species [1]–[3]. Insult or injury to the brain during this time may have significant consequences, manifested as behavioural changes due to the modifications in brain structure and function [4], [5]. Indeed, prenatal exposure to either environmental stress [6] or chemical toxins [7] may produce long-lasting behavioural changes. Thus, rats exposed to neurotoxic compounds during the first few weeks of postnatal development display spontaneous recurrent seizure activity upon reaching adulthood, evident both behaviourally and in electroencephalographic analyses [8], [9]. Exposure to such toxins also provokes the formation of a hyperexcitable hippocampal network *in vitro* [10], [11]. Similarly, deprivation of maternal care during the first week of life provokes emotional and cognitive behavioural alterations in rodents that are ultimately manifested in adults [12].

Proteins are the motors of virtually all biological processes, and the finely tuned equilibrium between their synthesis and degradation influences cellular homoeostasis. Accordingly, the deregulation of protein clearance and synthesis contributes to cell senescence, aging and various age-related disorders in the central nervous system [13]–[17]. Proteins that are of no use to a cell are disposed of through the ubiquitin proteasome system (UPS) [18], including mutant, misfolded, damaged, terminally modified or over-accumulated proteins [19]. The proteasome also degrades proteins involved in cellular processes such as signal transduction, cell-cycle regulation, metabolism, inflammation and apoptosis [20]–[22]. There is growing evidence that protein degradation has a strong influence on both neuronal development and long-term synaptic plasticity [23]–27. Indeed, the abnormal protein aggregates observed in many neurodegenerative diseases, such as Alzheimer's, Huntington's and Parkinson's diseases, reflect the dysfunctional protein degradation in these pathologies [28], [29].

In the last decade, considerable research has focused on the role of the UPS in aging, neurodegenerative diseases and synaptic plasticity. Here we focus on the role of the UPS during early postnatal mouse development and on the consequences of postnatal proteasome inhibition in later life. To address these issues, we administrated proteasome inhibitors in the temporal window from 1 to 10 days life as in this period the blood brain barrier is not completely closed and as such, proteasome inhibitors can reach the brain easily. Subchronic administration of the proteasome inhibitors MG132 or lactacystin provoked a significant decrease in chymotrypsin activity, an accumulation of ubiquitinated proteins, a decrease in mitochondrial markers and of their activity, and an increase in DNA oxidation. However, proteasome inhibition caused no behavioural or morphological alterations within this timeframe. Nevertheless, upon reaching 3–5 months of age, mice treated postnatally with proteasome inhibitors displayed impaired hippocampal- and amygdala-cognition, and mild motor dysfunction. These behavioural deficiencies were correlated with neuronal loss in the hippocampus, amygdala and brainstem, as well as diminished neurogenesis. Accordingly, these results indicate that decreased UPS function during early postnatal development provokes an acceleration of several features associated with aging, including neuronal degeneration, as well as motor and cognitive impairment, inducing a phenotype similar to that found in mouse models of mental retardation.

## Results

### Systemic administration of proteasome inhibitors in neonates decreases proteasomal and mitochondrial activity, and it increases DNA oxidation in the mouse brain, without affecting early psychomotor development

**Figure 1:**
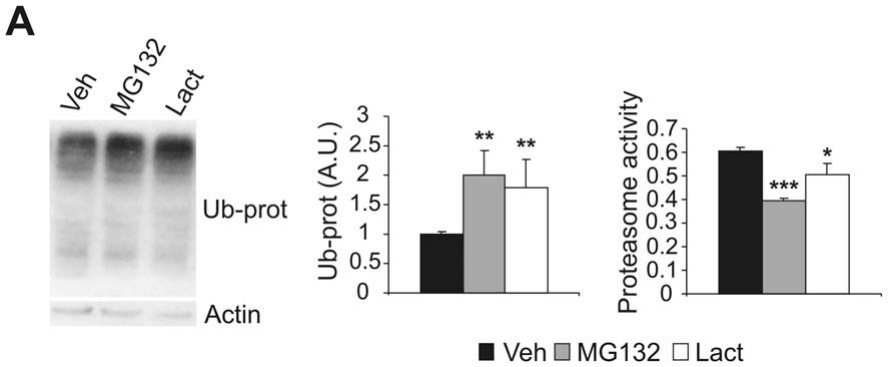
Proteasome inhibition during early postnatal development provokes the accumulation of ubiquitinated proteins. Mouse pups were treated with vehicle, MG132 or lactacystin for the first 10 days of life. One hour after the last injection, pups were sacrificed and total brain protein was extracted. **A** Representative western blot probed for ubiquitin and optical densitometry quantification of ubiquitinated proteins in vehicle-, MG132- and lactacystin-injected pups (left and right panels, respectively). **B** Chymotrypsin proteasome activity in control, MG132 and lactacystin treated pups (black, gray and white bars, respectively). n = 5 per group. *, p<0.05, **, *p*<0.01, ***, *p*<0.001.

To study the effect of early postnatal proteasome inhibition on adult mice during the first 10 days of life, we administered daily systemic injections of a proteasome inhibitor, MG132 (2.5 mg/kg) or lactacystin (1 mg/kg). To quantify the effect of proteasome inhibition on ubiquitinated proteins, western blots of total brain protein from PD10 mice were probed with an antibody against ubiquitin. Ubiquitinated proteins were more abundant in brain extracts from MG132- and lactacystin-treated mice than in their respective controls (MG132: t(6) = 4.679, *p* = 0.003; Lactacystin: t(6) = 4.605, *p* = 0.004. **Fig. 1A**). To confirm that the proteasome had been inhibited, chymotrypsin proteasomal activity was measured in the brains of PD10 mice treated with proteasome inhibitors. Both MG132- and lactacystin-treated mice exhibited a significant decrease in chymotrypsin activity when compared with the control mice (MG132: t(9) = 8.456, *p*<0.001; Lactacystin: t(9) = 2.814, *p* = 0.002. **Fig. 1B**). Hence, the two proteasome inhibitors used appear to reach the brain and effectively inhibit proteasome activity.

**Figure 2:**
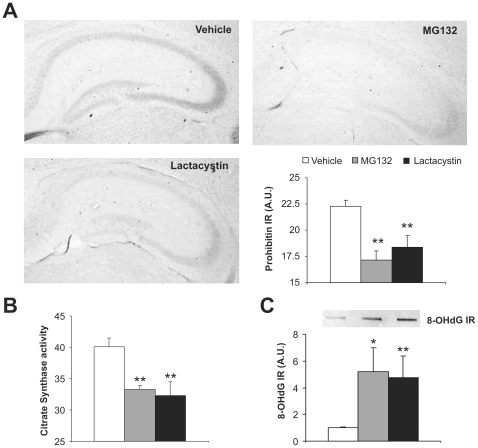
Proteasome inhibition during early postnatal development provokes a decrease in the expression of mitochondrial markers and an increase in DNA oxidation. Mouse pups were treated with the vehicle alone, MG132 or lactacystin for the first 10 days of life and 1 hour after the last injection, the pups were killed and their brains removed. **A** Representative immunohistochemistry and optical densitometry quantification of prohibitin in pups treated with the vehicle alone, MG132 or lactacystin (black, gray and white bars, respectively). **B** Citrate synthase activity in brain protein extracts from control, MG132 or lactacystin treated pups. **C** Representative dot blot and optical densitometry quantification for 8-OH-deoxyguanine (8-OH-dG), a marker of DNA oxidation, in total DNA extracted from the brains of control, MG132 or lactacystin treated mice. n = 5 per group. *, *p*<0.05; **, *p*<0.01.

We investigated the effects of proteasome inhibition on other molecular processes, focusing initially on the mitochondria as there is evidence that mitochondrial quality is controlled by the ubiquitin-proteasome system [30], [31]. Early postnatal inhibition of proteasome activity diminished the amount of prohibitin detected by immunohistochemistry, an integral protein of the inner mitochondrial membrane (22.26±0.6, 17.17±0.87 and 18.36±1.13 arbitrary units in control, MG132 and lactacystin-treated mice respectively: F(10, 2) = 12.21, *p* = 0.002. **Fig. 2A**). Similarly, citrate synthase activity was also reduced (40.05±1.39, 33.25±0.67 and 32.31±2.22 µg/mg in control, MG132- and lactacystin-injected mice, respectively: F(21, 2) = 8.71, *p* = 0.001. **Fig. 2B**). Together, these results suggest that the administration of proteasome inhibitors decreased the number and/or activity of mitochondria, an effect that would probably induce oxidative stress [32]. Hence, we assessed the oxidative stress in these mice by evaluating the presence of 8-OH-deoxyguanine (8-OH-dG) in the total DNA extracted from control, MG-132- and lactacystin-injected mouse brains. Proteasome inhibition during early postnatal development augmented 8-OH-dG immunoreactivity (5.18±1.8 in MG132- and 4.73±1.64 fold in lactacystin-treated mice relative to the controls: F(19, 2) = 4.36, *p* = 0.02. **Fig. 2C**). These results suggest that proteasome inhibition during early postnatal development provokes mitochondrial dysfunction and increased oxidative stress.

To determine the effect of proteasome inhibition on the postnatal psychomotor development of mice, we performed behavioural tests included in the Fox's battery daily from PD 1 to 15 (**Fig. S1**). In terms of balance reflexes (measured through the righting reflex, cliff drop aversion, negative geotaxia or the suspension test: **Fig. S1B, C and E**) or locomotor activity (pivoting activity test: ****), there were no significant differences between mice injected with proteasome inhibitors and control mice during the first two weeks of life. Moreover, while some differences in the body weight of MG132-treated mice were observed initially (**Fig. S1A**), these disappeared later with age. When brain morphology was analyzed by staining with antibodies against calbindin, tyrosine hydroxylase and doublecortin, there were no significant differences in the hippocampus, cerebellum, amygdala or substantia nigra of PD15 mice treated with proteasome inhibitors when compared to their control littermates (**Fig. S2**).

### Proteasome inhibition during early postnatal development induces mild alterations in exploratory activity in adult mice

**Figure 3:**
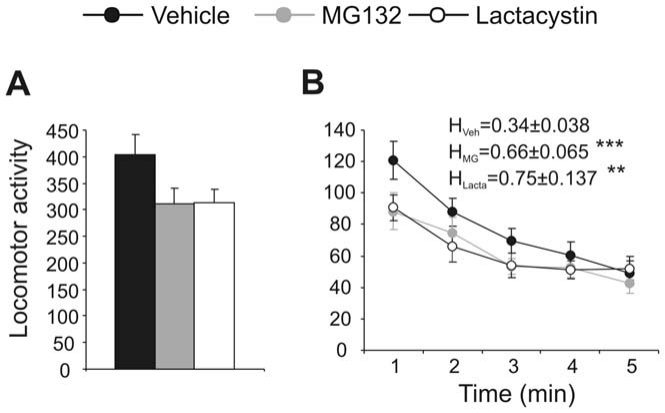
Effect of postnatal proteasome inhibition on exploratory activity in adult mice. **A** Total motor activity of mice treated with the vehicle alone, MG132 and lactacystin (black, gray and white symbols, respectively), as measured in an open field for 5 minutes. **B** Minute-by minute evolution of motor activity over 5 minutes in the same experimental animals. The habituation index was determined as the ratio between the activity in the last and the first minute in the open field. n = 15, 10 and 10 in control, MG132, and lactacystin treated groups, respectively. **, *p*<0.01; ***, *p*<0.001.

To determine whether temporary inhibition of proteasome activity during the first 10 days of life produces behavioural alterations in adult mice, exploratory activity in 3–5 month old mice was evaluated over 5 minutes in the open field test. Mice treated with proteasome inhibitors exhibited a mild non-significant decrease in locomotor activity when compared with control mice (**Fig. 3A**). The capacity to habituate to a new environment was evaluated as the ratio of activity in the first and last minute in the open field (**Fig. 3B**). This habituation index (H) was higher in mice treated postnatally with proteasome inhibitors than in control mice (MG132: t(23) = 0.041, *p* = 2.15; lactacystin: t(23) = 0.032, *p* = 2.26). These findings suggest that proteasome inhibition during early postnatal development provokes cognitive deficiencies in adulthood.

### Proteasome inhibition during early postnatal development results in mild symptoms of depression, decreased co-ordination and a loss of brainstem dopaminergic neurons in adult mice

**Figure 4:**
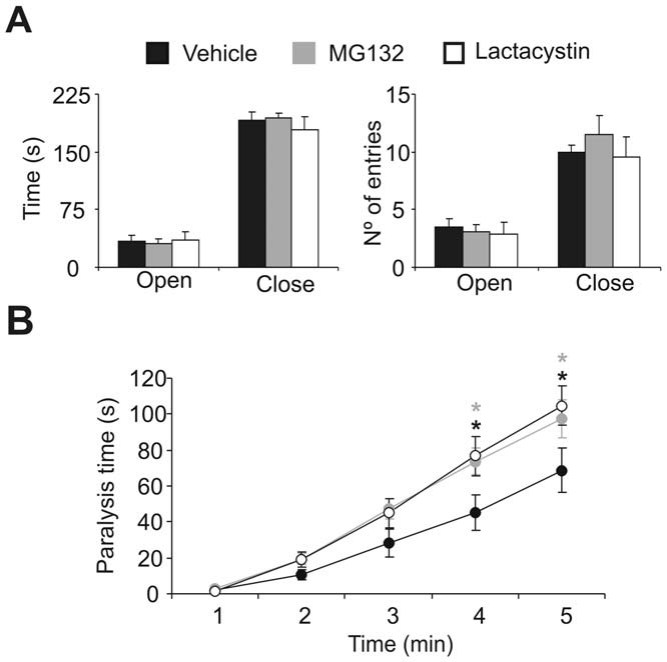
Proteasome inhibition during early postnatal development induces depression-like symptoms in adult mice. The behaviour of mice treated postnatally with proteasome inhibitors was assessed in the elevated plus maze (A) and tail suspension test (B). **A,** The time (s) and number of entries into the open and closed arms was measured over 5 minutes. **B,** Accumulated time spent immobile (s) over 5 minutes in the tail suspension test. Black, gray and white bars or circles represent mice treated with the vehicle alone (n = 15), MG132 (n = 10) and lactacystin (n = 11), respectively. *, *p*<0.05.

**Figure 5:**
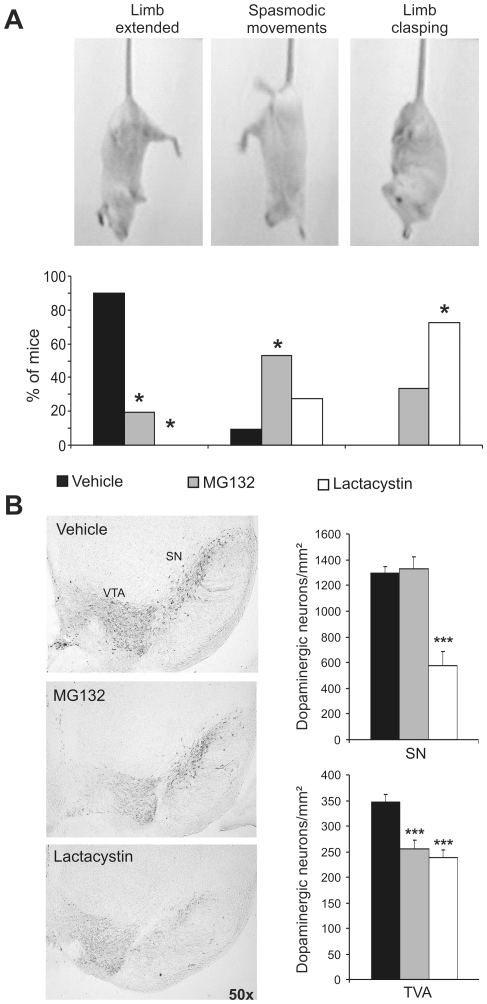
Proteasome inhibition during early postnatal development provokes discoordination and a loss of brainstem dopaminergic neurons in adult mice. **A** (Upper panel) photograph showing examples of limb extension, spasmodic movement and limb clasping during the tail suspension test. Bar graph represents the percentage of mice exhibiting each behaviour in the tail suspension test for each experimental group. Black, gray and white bars represent mice treated with the vehicle alone (n = 14), MG132 (n = 15) and lactacystin (n = 11), respectively. **B** Representative photomicrograph showing brainstem dopaminergic neurons in mice treated with the vehicle alone, MG132 and lactacystin. Bar graph showing the density of dopaminergic neurons (neurons/mm^2^) in the susbtantia nigra (SN) and ventral tegmental area (VTA) in the same experimental groups as those shown in A. n = 8 mice per group. *, *p*<0.05; ***, *p*<0.001.

To study cognition in adult mice subjected to postnatal proteasome inhibition, 3–5 month old mice were subjected to tests of anxiety-like behaviour in the elevated plus maze, which revealed no differences between mice treated with proteasome inhibitors and their controls (**Fig. 4A**). Moreover, immobility in the tail suspension test was used to measure depression-like behaviour, which revealed increased immobility in mice treated with proteasome inhibitors when compared to their controls (**Fig. 4B**). The tail suspension test can also reveal balance impairment that may result from a loss of brainstem dopaminergic neurons. Interestingly, more of the mice treated with proteasome inhibitors as neonates exhibited spasmodic movements (53.3% and 27.27% of MG132- and lactacystin-injected mice, respectively) and limb clasping (33.3% and 72.72% of MG132- and lactacystin-injected mice, respectively: **Fig. 5A**). As this abnormal motor behaviour could be attributed to the motor dysfunction found in mouse models for motor neuron diseases, we performed specific behavioural tests for motor dysfunction: Rotarod, treadmill, and grip strength (**Fig. S3A–C**). No significant differences were found in vehicle-, MG132- and lactacystin-injected mice in any of these tests. Furthermore, when we studied the histology of the spinal cord, there were no differences between these three experimental groups (**Fig. S3D**). Hence, mice treated with proteasome inhibitors during postnatal development appear to suffer depression and altered motor coordination in adulthood.

Given the role of brainstem, substantia nigra (SN) and ventral tegmental area (VTA) dopaminergic neurons in regulating movement co-ordination, we assessed the levels of tyrosine hydroxylase (TH) protein by immunohistochemistry. Fewer TH^+^ neurons were detected in the SN and VTA of adult mice treated postnatally with lactacystin than in control mice (SN: t (15) = 4.351, *p*<0.001; VTA: t (15) = 6.846, *p*<0.001. **Fig. 5B**). Similarly, adult mice treated neonatally with MG132 had fewer TH^+^ neurons in the VTA (t (15) = 3.954, *p*<0.001). These decreases in the number of TH^+^ neurons in the SN and VTA were probably correlated with the severity of the impairments in their co-ordination.

### Adult mice treated with proteasome inhibitors during early postnatal development exhibit memory defects and neuronal loss in the hippocampus and amygdala

**Figure 6:**
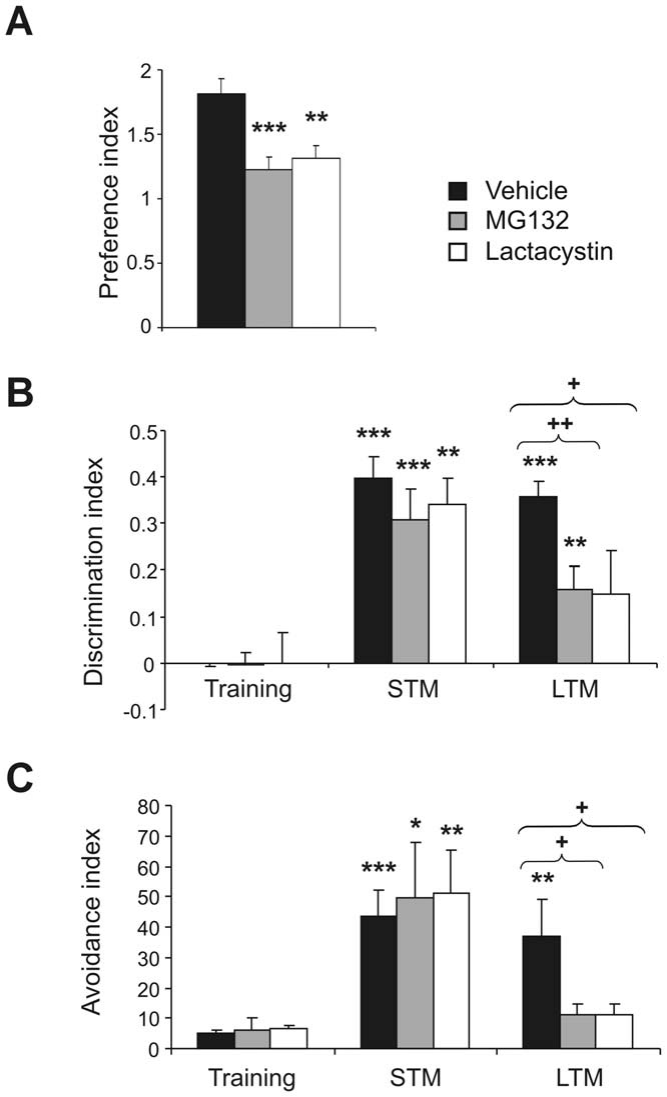
Memory deficiencies in adult mice treated postnatally with proteasome inhibitors. Cognitive capacity was assessed in adult mice treated postnatally with proteasome inhibitors using the Y maze (A), object recognition test (B) and passive avoidance test (C). **A,** New arm preference in the Y maze was assessed during a short term memory test performed 2 minutes after a 10 minute training session (n = 16, 15 and 11 in control, MG132 and lactacystin treated animals, respectively). **B,** Discrimination index in short- and long-term (STM and LTM) memory training sessions in the object recognition test (n = 14, 11 and 6 in control, MG132 and lactacystin treated animals, respectively). **C,** Avoidance index during STM and LTM training sessions in the hot plate passive avoidance test (n = 8 mice per group). * indicates significant differences between the STM or LTM session compared with the training session; + indicates significant differences between groups in the same session. Black, gray and white bars represent mice treated with the vehicle alone, MG132 and lactacystin, respectively. * or ^+^, *p*<0.05; ** or ^++^, *p*<0.01; ***, *p*<0.001.

To identify possible cognitive deficiencies in adult mice treated postnatally with proteasome inhibitors, as suggested by the exploratory habituation findings, we performed several learning and memory tests that depend on different brain areas. In the hippocampal-dependent Y-maze, we evaluated the preference of the mice for the novel arm of the maze. Animals treated with proteasome inhibitors at early ages showed a weaker preference for the novel arm than control mice (1.88±0.11, 1.22±0.1 and 1.31±0.03 for vehicle-, MG132- and lactacystin-treated mice, respectively: F(39, 2) = 11.46, *p*<0.001. **Fig. 6A**). The object recognition memory test, a one trial test also dependent on hippocampal function, revealed that adult mice treated postnatally with proteasome inhibitors had impaired long-term memory (LTM), evaluated 24 hours after the training session (the discrimination indices were 0.35±0.03, 0.15±0.04 and 0.15±0.09 in vehicle-, MG132- and lactcystin-treated mice respectively: F(28, 2) = 6, *p* = 0.006. **Fig. 6B**). By contrast, no effects on short-term memory (STM) were observed 1 hour after the training session (F(28, 2) = 0.71, *p* = 0.49). Furthermore, proteasome inhibitor treatment failed to alter the exploration times with respect to the vehicle treated group in the OR test (Table S1). Finally, mice were subjected to the hot plate passive avoidance test, an amygdala-dependent learning and memory test. The avoidance index for STM revealed that all experimental animals learned the task irrespective of the treatment administered (43.34±8.88%, 49.41±19.25%, and 51.02±14.16% for mice treated with the vehicle alone, MG132 and lactacystin, respectively: F(21.2) = 0.08, *p* = 0.92. **Fig. 6C**). However, adult mice treated postnatally with proteasome inhibitors had a lower avoidance index in the LTM test than the control mice (42.36±12.05%, 11.33±3.32%, and 11.35±3.43% mice treated with the vehicle alone, MG132 and lactacystin, respectively: F(21,2) = 5.72, *p* = 0.01).

**Figure 7:**
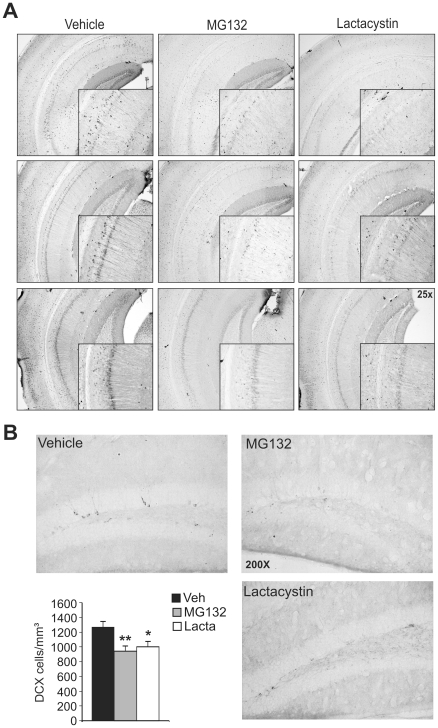
Hippocampal neuronal loss and deficient neurogenesis in adult mice treated postnatally with proteasome inhibitors. **A,** Hippocampal integrity was assayed by calbindin immunohistochemistry. Representative photomicrograph showing different antero-posterior hippocampal levels in adult mice treated postnatally with the vehicle alone, MG132 or lactacystin (n = 8 per group). **B,** Neurogenesis was evaluated by doublecortin (DCX) immunohistochemistry. Representative photomicrographs of DCX immunoreactivity in the hippocampal dentate gyrus are shown and the histograms show the stereological quantification of DCX^+^ neurons in the subgranular layer of the dentate gyrus. Black, gray and white bars represent mice treated with the vehicle alone, MG132 and lactacystin, respectively (n = 8 per group). *, p<0.05, **, *p*<0.01.

As the hippocampus and amygdala play central roles in learning acquisition and memory consolidation in the tests used, these structures were analysed morphologically by calbindin immunohistochemistry. In both structures, fewer neurons expressing calbindin were observed in adult mice treated postnatally with proteasome inhibitors when compared to the controls (**Fig. 7A** **and** **8**). As adult hippocampal neurogenesis (AHN) appears to be involved in behavioural alterations and neuronal plasticity, we analyzed neurogenesis by doublecortin (DCX) immunohistochemistry. The number of neurons in the hippocampus labelled for DCX was significantly lower in adult mice treated postnatally with proteasome inhibitors than in control animals (**Fig. 7B**). These results suggest that mice treated with proteasome inhibitors during postnatal development experience premature neuronal loss in the hippocampus and amygdala, which may account for the impaired cognitive abilities observed.

**Figure 8:**
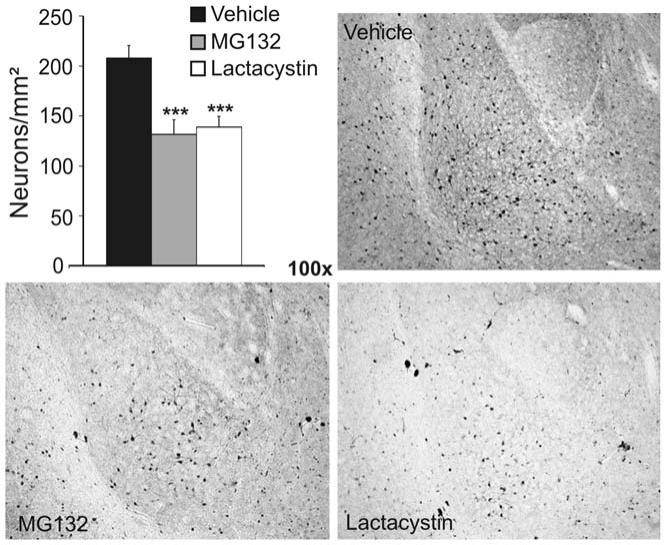
Proteasome inhibition during early postnatal development provokes a loss of calbindin neurons in the amygdala of adult mice. Amygdala integrity was assayed by calbindin immunohistochemistry (Representative photomicrograph showing the amygdala of adult mice treated postnatally with the vehicle alone, MG132 or lactacystin). Bar graph shows neuronal density (neurons/mm^2^) in the amygdala in each experimental group. Black, gray and white bars represent control, MG132 and lactacystin treated mice, respectively (n = 8 mice per group). ***, *p*<0.001.

## Discussion

The UPS provides a useful mechanism to dispose of biologically compromised proteins in a cell, including mutant, misfolded, damaged, terminally modified or over-represented proteins [19]. In addition to this waste basket-like function, the UPS provides a highly specific means of controlling the expression of proteins involved in signal transduction pathways, in cell–cell communication during neuronal development, in nerve transmission at the neural synapse and in long-term synaptic plasticity 23–26,33,34. Abnormal protein aggregates, including those containing polyubiquitinated protein, are associated with aging and with many neurodegenerative diseases, such as Alzheimer's, Huntington's and Parkinson's diseases, suggesting that UPS dysfunction may be implicated in these diseases [28], [29]. Although much research has focused on the role of proteasome dysfunction in aging and neurodegenerative diseases, the consequences of postnatal inhibition of the UPS in adult mice have been little studied. As such, we focused on the role of the proteasome during early postnatal development (PD 1–10) and the consequences of its inhibition during this period in adult mice. Interestingly, administering proteasome inhibitors during early development caused no immediate alterations in behaviour that could be detected in the Fox test. However, upon reaching adulthood, mice treated postnatally with proteasome inhibitors exhibited a complex behavioural phenotype, with features of depression, motor discoordination, and cognitive alterations in several learning and memory tests. Many of these behaviours are also associated with neurodevelopmental and mental retardation disorders.

At the cellular level, motor co-ordination and cognitive deficits are linked to functional impairment in specific brain areas. Motor co-ordination is primarily linked to the brainstem, striatum and cerebellum, as evident through the characteristic motor disturbances associated with neurodegeneration in these areas in Parkinson's and Huntington's diseases, and in ataxia [35]–[37]. Morphological analysis of the brains of adult mice treated postnatally with proteasome inhibitors revealed a decrease in the density of dopaminergic neurons in the brainstem. Furthermore, the severity of motor discoordination was correlated with the loss of these dopaminergic neurons. Indeed, dopaminergic neurons were lost in both the substantia nigra and VTA of lactacystin treated mice, while only the latter was affected in MG132 treated mice. A decrease in the number of calbindin positive neurons was also observed in the hippocampus and amygdala of mice treated postnatally with proteasome inhibitors, both these regions playing a critical role in different learning and memory tasks: the amygdala is involved in emotional cognition [38], [39]; and the hippocampus in spatial and associative cognition [40], [41]. Interestingly, postnatal proteasome-inhibition impaired long-term memory as evident in the object recognition and the passive avoidance test. By contrast, alterations to short-term memory following proteasome inhibition were observed in open-field habituation and the Y maze, tests of hippocampal function [42]. Finally, postnatal proteasome inhibition also produced depression-like symptoms in adulthood, as well as a decrease in AHN. A correlation between neurogenesis and depression has already been described [43]–[45], and AHN has been linked with learning consolidation [46]. Hence, a decrease in the neurogenic response provoked by proteasome inhibition could contribute to the impaired learning consolidation. Together, these findings suggest that morphologically undetectable alterations in neonatal brain development induced by proteasome inhibition may form the basis of a variety of different neurological pathologies in adults. In agreement, several neurodevelopmental diseases, mental retardation and schizophrenia, have all been linked to proteasome deficiencies [47].

The early postnatal period is a critical stage of brain development during which excitatory and inhibitory functions in the brain are organized and mature. For this reason, environmental stress or exposure to excitatory toxins during this period can provoke behavioural alterations in adulthood [6], [48], [49]. The cellular and molecular bases of these long-term effects remain poorly understood. Reduced maternal care has been shown to provoke persistent alterations in gene expression, probably due to long-term modifications in gene methylation profiles [12], [50], and these alterations are correlated with depressive-like behaviour in later life. In the present study, we demonstrate that postnatal proteasome inhibition causes long-term behavioural and morphological alterations in adult mice. These alterations may result from a decrease in mitochondrial activity together with an increase in DNA oxidation, both of which appear to be associated with proteasome inhibition in the brain of PD15 mice. Proteasome inhibition reduces complex I and complex II activity, and intra-mitochondrial protein translation, while it increases the production of mitochondrial reactive oxygen species. In addition, impaired UPS activity alters mitochondrial DNA and impairs mitochondrial turnover [51], [52]. Any or all of these events may occur in the postnatal brain in response to proteasome inhibition, representing the starting point for the neurodegeneration observed in the subtantia nigra, amygdala and hippocampus when these animals reach adulthood. Our results are supported by similar findings in the adult brain of conditional psmc1 knockout mice, in which the activity of the 26S proteasome is impaired. In these mice, deficiencies in proteasome activity result in early and lethal neurodegeneration, beginning at 3–4 weeks of age [53].

Proteasome dysfunction is considered a key contributor to aging-associated neurodegeneration. Indeed, brains from aging individuals and/or those suffering neurodegeneration exhibit clear deficiencies in proteasome activity. Furthermore, mutations in some of the components of the proteasome pathways have been linked to neurodegeneration, mental retardation and other neurodevelopmental diseases. Together, our results demonstrate that subchronic proteasome inhibition during early postnatal development, probably through mitochondrial dysfunction, triggers the development of a complex behavioural phenotype that is correlated with degeneration in specific brain areas. This phenotype shares many features with mental retardation syndromes, suggesting that proteasome dysfunction plays a critical role in the development of these syndromes.

## Materials and Methods

### Animals

Swiss mice weighing 25–30 g were obtained from University of Seville animal facilities and used as the parental mice. The mice were maintained under standard housing conditions, at 21±1°C with a photoperiod of 12:12 h (lights on at 07:30 h), and using dust-free sawdust. Food pellets and water were available *ad libitum*. Breeding pairs were formed and females were isolated when visibly close to parturition. Litters were adjusted to a maximum of ten pups in order to avoid any effects of parity on behavioural ontogeny [54].

### General procedure for postnatal observations

All pregnant dams were allowed to deliver spontaneously. The day of birth was designated as postnatal day (PD) 0 (±6 h). On delivery, the litter size of each dam was recorded and each pup was checked for gross abnormalities. Pups were individually marked with India ink on PD1 and nursed by their natural dams until weaning. From PD 1 to 10 (inclusive), the pups were weighed and subcutaneously administered proteasome inhibitors daily (2.5 mg/kg of MG132 or 1 mg/kg of lactacystin, injected under the skin back) or the vehicle alone (DMSO for MG132 or saline for lactacystin). After weaning, the mice were allowed to grow to 3–5 months of age in groups of 4–5. Upon reaching adulthood (from 3 to 5 months old), cognitive behavioural tests were performed. The sex distribution of the treatments was random and while histological analysis at P15 was performed mainly on female mice, adult behaviour and histology were performed on male mice in order to avoid the oestrus cycle. All experimental protocols were approved by the Ethics Committee of the Pablo de Olavide University (07/4-20/12/2008), in accordance with the European Community guidelines (86/609/EEC amended by Directive 2005/65/EC) and the Spanish regulations for the procurement and care of experimental animals (1201 RD/2005, October 10).

### Neonatal behavioural tests

During the testing protocols included in the Fox battery [55], whole litters were separated from the dams and maintained for 30 min in a warm environment. Males and females were pooled for neurodevelopmental screening after preliminary experiments demonstrated that there was no significant effect of sex. The range of ages at which responses were observed was evaluated in preliminary experiments in order to define the period of observation and to reduce handling. All testing was performed between 07:30 a.m. and 12:00 a.m. After completing the experimental procedure with each litter, the mice were weighed, injected and returned to the mother. The neonatal behavioural tests are described in **Methods S1**.

### Behavioural testing of adult mice

Behavioural tests were performed in a room with homogeneous sound and light. To eliminate any odours or traces that might affect the outcome of the test, the behavioural apparatus was cleaned with 70% ethanol (Panreac Química S.A.U) between the testing of each animal. The order in which the tests were run was always the same: Open field, object recognition memory, Y maze, plus maze, tail suspension and step down passive avoidance.

#### Motor activity in the open field

To evaluate locomotor and exploratory activity, mice were placed for 5 minutes in an open field (38×21×15 cm) (Cybertec S.A.). This apparatus consisted of a walled platform containing infrared emitters and sensors (IR) coupled to an actimeter, and the movement sensor was connected to a computer that recorded the number of times the mouse interrupted the IR beams/min.

#### Elevated plus maze

The elevated plus maze consisted of two open and two closed arms of equal dimensions extending from a central area, and it was elevated 70 cm above the ground. Mice were placed at the end of the open arm in brightly lit conditions and allowed to explore the maze for 5 minutes. The percentage of entries and the time spent in the open and closed arms were measured.

#### Y maze

The Y maze used had three equal sized arms (8×40×20 cm), the walls of which were decorated with different patterns to enable the mice to discriminate between the arms. Mice were allowed to freely explore two arms during a 10 minute training session, after which they were returned to their cages for 2 minutes while olfactory cues were cleaned from the maze. Subsequently, mice were returned to the maze and allowed to explore all three arms for 10 minutes. Short-term memory was determined as the number of entries into the new arms/average number of entries into the familiar arms.

#### Object recognition memory

Mice were tested in a rectangular arena (55×40×40 cm) located in a room with dim lighting and constant background noise. For the object recognition protocol, two different objects were placed in the arena during the training phase. The animal's memory of the original object was assessed by comparing the amount of time spent exploring the novel object against that for the familiar one. The objects consisted of plastic pieces of different shapes and they were cleaned thoroughly with 70% ethanol between trials to remove any olfactory cues. Before the experiment, mice were habituated to the arena for 20 min in the absence of the objects on 2 consecutive days. On the day of training, mice were allowed 15 min to explore the two objects. Retention tests were then performed at the times indicated after the training session by placing the mice back in the arena for a 10 min session after randomly exchanging one of the familiar objects for a novel object. The time spent exploring each object was recorded and the relative exploration of the novel object was expressed by a discrimination index [DI = (t_novel_−t_familiar_)/(t_novel_+t_familiar_)]. The criteria for exploration were based strictly on active exploration. Exploration of an object was defined as directing the nose toward the object at a distance of ±1.5 cm and/or touching the object with the nose or vibrissae. Circling or sitting on the object were not considered exploratory behaviour. All trials were performed by an experimenter blind to the drug treatments.

#### Step down passive avoidance

This learning test was performed on a hot plate placed inside a rectangular chamber (15×15×21 cm), which contained a step that allowed the mice to avoid the negative stimulus (a heat shock of 60±0.5°C). After a habituation session in the absence of a negative stimulus, mice were subjected to a training session in which they were placed on the step and the time taken to descend was determined. Each session lasted 30 seconds. By leaving the animal on the platform and quantifying the time taken to step off in the absence of a heat stimulus, short- and long-term memory were evaluated 10 minutes and 24 hours after finishing the training session, respectively. The response inhibition was calculated as the percentage of the total time the mice spent on the step.

#### Tail suspension

Mice were suspended above the floor by fixing the end of the tail to wire netting and immobility was scored by manual observation during a 5 min test session. Motor co-ordination was also scored, as revealed by the movements made to maintain balance (*i.e.*: spasmodic movements or limb clasping).

### Tissue Preparation and Immunohistochemistry

To quantify the morphological alterations in the brain induced by treatment with proteasome inhibitors, mice from each experimental group were sacrificed by decapitation, and their brains were removed and fixed by immersion for 24 h at 4°C in 4% paraformaldehyde prepared in phosphate-buffered saline (PBS). The tissue was cryoprotected in 30% sucrose-PBS for 2 days at 4°C and coronal brain sections (50 µm) were then processed for free-floating immunohistochemistry [56] using specific antisera against: calbindin (Swant Antibodies, diluted 1∶3,000), tyrosine hydroxylase (TH, Pel-Freez Biologicals, diluted 1∶3,000), doublecortin (DCX, Santa Cruz Biotechnologies, diluted 1∶1,000), and prohibitin (Epitomics, diluted 1∶500). Densitometry and quantification of the neurons in the sections were performed using Image-J software (downloaded as a free software package from the public domain: http://rsb.info.nih.gov/ij/download.html). For DCX immunostaining we performed stereological quantification using an optical dissector to measure cell density. The volume of the subgranular layer of the dentate gyrus was measured by the standard Cavalieri method with the aid of ImageJ. By multiplying the mean cell density and the total reference volume for each animal, the total number of cells per animal was obtained.

### Protein extraction

Fresh brain areas were dissected at 4°C and homogenized by sonication for 15 seconds in 1 mL of cold lysis buffer: 10 mM Tris-HCl (pH 7.8), 0.5 mM DTT, 5 mM MgCl_2_. The lysates were centrifuged at 400×g for 10 minutes at 4°C, 20% glycerol was added to the protein supernatant obtained and the samples were stored at −80°C. Protein quantification was performed using the BioRad protein assay (Bio-Rad Laboratories, Inc).

### Proteasome activity assay

The fluorogenic substrate Succ-LLVY-AFC (N-Succinyl-Leu-Leu-val-Tyr 7-Amido 4trifluormethylcoumarin, Sigma Aldrich) was used to measure chymotrypsin-like proteasome activity. Assays were carried out at 37°C in a 50 µl reaction volume containing: 12.5 µg protein extract, 5 mM ATP (AppliChem GmBH), 50 mM EDTA (Sigma), and 5 µM Succ-LLVY-AFC in lysis buffer. The rate of cleavage of the fluorogenic peptide substrates was determined in a multiwell plate reader (Varioskan flash-Termo Fisher Scientific) by monitoring the fluorescence of the fluoromethylcoumarin released every 5 minutes over 1 hour, with an excitation wavelength of 395 nm and emission wavelength of 460 nm. Proteasome activity was expressed as the slope of the enzymatic kinetics.

### Measurement of citrate synthase activity

The specific activity of citrate synthase in cell extracts prepared from brain tissues was measured at 412 nm minus 360 nm (13.6 mmol/L/cm) using 5,5-dithio-bis(2-nitrobenzoic acid) to detect free sulfhydryl groups in coenzymeA, as described previously [57].

### Immunoblotting

For dot blots, 5 µg of DNA from mouse brains was digested with EcoRI and heat denatured. DNA was diluted in 200 µl of 6× SSC and transferred to a nitrocellulose membrane using a Bio-Dot SF Microfiltration Apparatus (Bio-Rad). Protein extracts from the areas selected were resolved on 10% polyacrylamide gels for SDS–PAGE electrophoresis and subsequently transferred to PVDF membranes. After blocking, the membranes were probed overnight at 4°C with a primary goat polyclonal antiserum against 8-hydroxy-2-deoxyguanosine (1∶500, Millipore) for dot blots, or with a primary polyclonal antiserum against ubiquitin (Dako) or actin (Santa Cruz Biotechnologies) diluted 1∶1,000. Antibody binding was visualized with the ECL plus kit (Amersham Biosciences).

### Statistical Analysis

Statistical analyses were performed using the SPSS package for Windows (SPSS, Chicago, IL). Unless otherwise indicated, the data are represented as the mean ± SEM. Two-way analysis of variance (ANOVA) was used to compare behavioural results from animals treated with the vehicle alone or the proteasome inhibitors. Chi-squared tests were used for statistical analysis in cases where the qualitative variables were compared.

## Supporting Information

Figure S1 **Postnatal proteasome inhibition had no effect on body weight or neurobehavioural activity.** **A** The body weight of animals treated with proteasome inhibitors evolved similarly to that of mice that received the vehicle alone, except for a small decrease in P9-10 mice treated with MG132 [t (28) = 2.233, P = 0.034], which was subsequently reversed. **B–E,** Fox Battery tests carried out in animals treated with proteasome inhibitors revealed no significant differences with respect to the group that received the vehicle alone in terms of the righting reflex (B), negative geotaxia (C), rotating activity (D) or muscle strength in the suspension test (E). Taken together these data, suggest that proteasome inhibition during early postnatal development does not affect the pup's behaviour.
(TIF)

Figure S2 **Postnatal proteasome treatment caused no morphological alterations at PD15.** At PD15, mice treated with proteasome inhibitors or the vehicle alone were sacrificed, and their brain histology was analysed. A–C Immunohistochemistry for calbindin revealed no differences in hippocampal dendritic morphology (A) in either the cerebellum (B) or amygdala (C) of mice treated with proteasome inhibitors, or with the vehicle alone. D Tyrosine hydroxylase immunohistochemistry revealed no differences in neuronal density in the *substantia nigra* of mice injected with proteasome inhibitors or the vehicle alone. E Neurogenesis in P15 mice was unaffected by postnatal proteasome inhibition, as revealed by doublecortin (DCX) immunostaining.
(TIF)

Figure S3 **Proteasome inhibition during early postnatal development does not induce motor behaviour or morphological alterations in the spinal cord of adult mice.** A–C, Motor function in adult mice that were treated with proteasome inhibitors during early life was measured by their performance in the rotarod (A), treadmill (B) and grip strength (C) tests. D, Representative Nissl staining microphotograph at lumbar 4–5 level of spinal cord is shown (D).
(TIF)

Table S1 **Total object exploration times (in seconds) of adult mice injected postnatally with the vehicle alone, MG-132 or lactacystin in each session of a 15-minute OR memory test.** STM, short-term memory; LTM, long-term memory.
(DOC)

Methods S1 **Detailed description of neonatal behavioural tests included in the Fox battery.**
(DOC)
