# Supplementary figures and images for: Postnatal Proteasome Inhibition Induces Neurodegeneration and Cognitive Deficiencies in Adult Mice: A New Model of Neurodevelopment Syndrome
Source: PLoS One. 2011 Dec 12;6(12):e28927. doi: 10.1371/journal.pone.0028927 (PMC3236230; doi:10.1371/journal.pone.0028927)

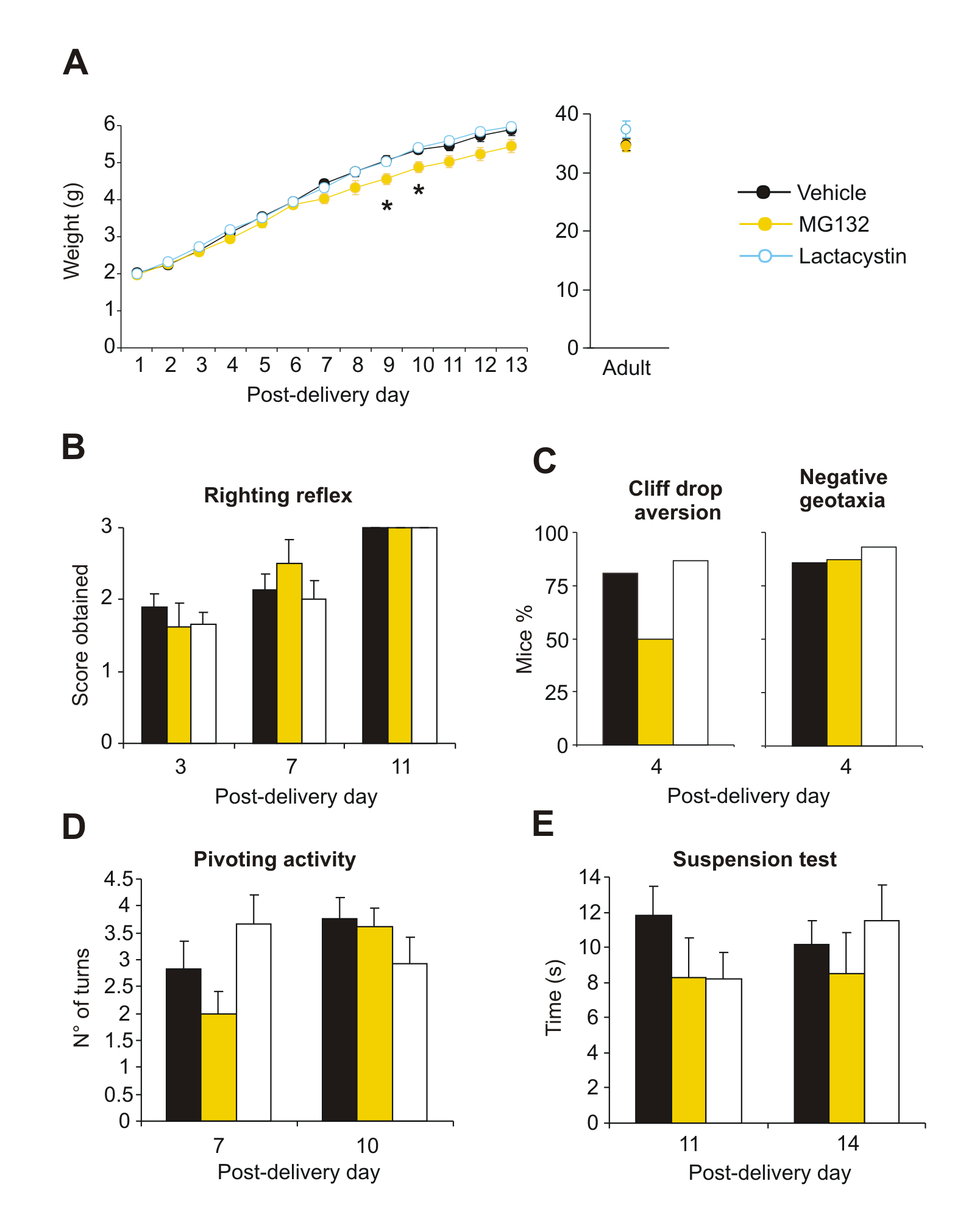

Supplement: Figure S1 — Postnatal proteasome inhibition had no effect on body weight or neurobehavioural activity. A The body weight of animals treated with proteasome inhibitors evolved similarly to that of mice that received the vehicle alone, except for a small decrease in P9-10 mice treated with MG132 [t (28) = 2.233, P = 0.034], which was subsequently reversed. B–E, Fox Battery tests carried out in animals treated with proteasome inhibitors revealed no significant differences with respect to the group that received the vehicle alone in terms of the righting reflex (B), negative geotaxia (C), rotating activity (D) or muscle strength in the suspension test (E). Taken together these data, suggest that proteasome inhibition during early postnatal development does not affect the pup's behaviour. (TIF) [file pone.0028927.s001.tif]

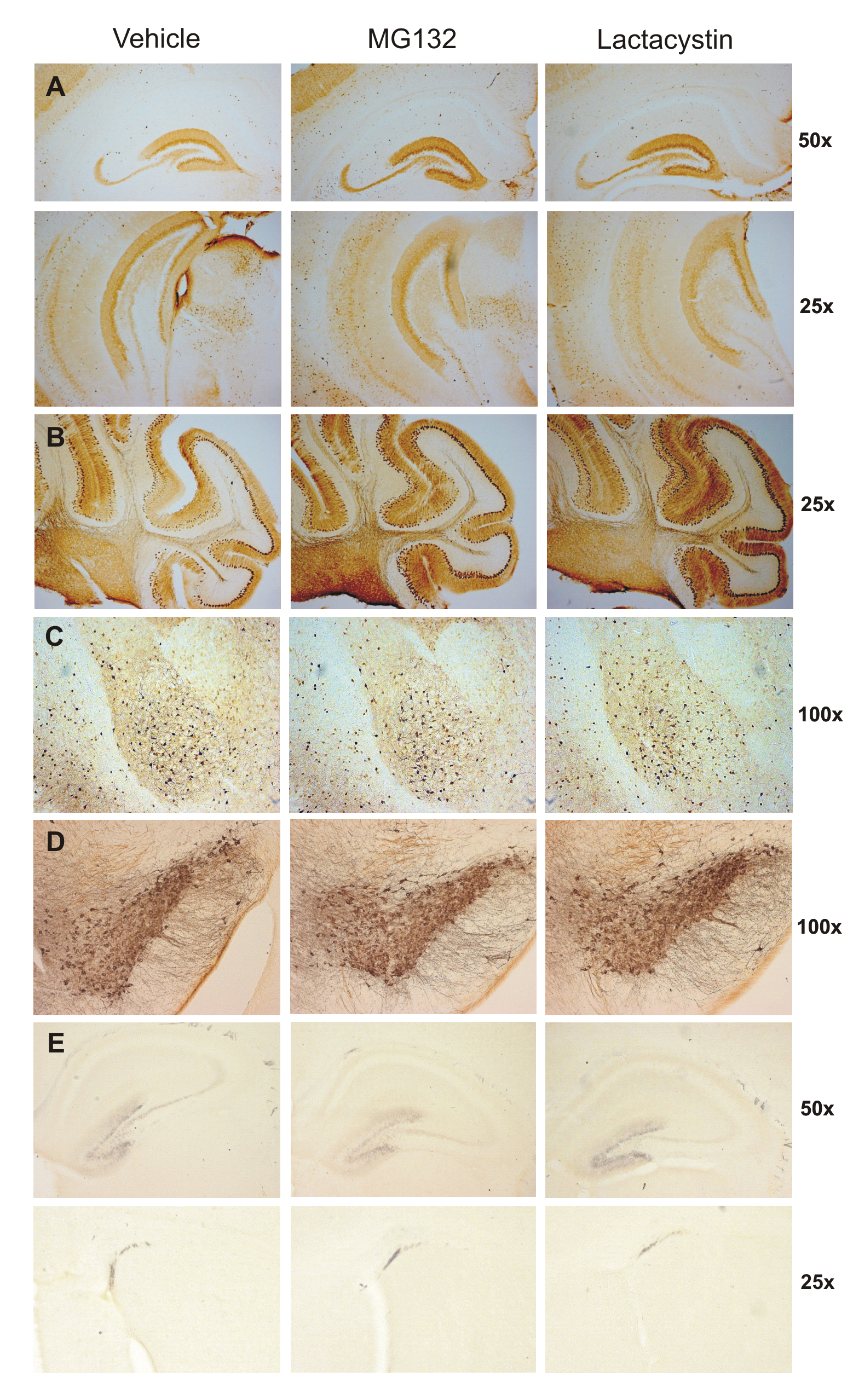

Supplement: Figure S2 — Postnatal proteasome treatment caused no morphological alterations at PD15. At PD15, mice treated with proteasome inhibitors or the vehicle alone were sacrificed, and their brain histology was analysed. A–C Immunohistochemistry for calbindin revealed no differences in hippocampal dendritic morphology (A) in either the cerebellum (B) or amygdala (C) of mice treated with proteasome inhibitors, or with the vehicle alone. D Tyrosine hydroxylase immunohistochemistry revealed no differences in neuronal density in the substantia nigra of mice injected with proteasome inhibitors or the vehicle alone. E Neurogenesis in P15 mice was unaffected by postnatal proteasome inhibition, as revealed by doublecortin (DCX) immunostaining. (TIF) [file pone.0028927.s002.tif]

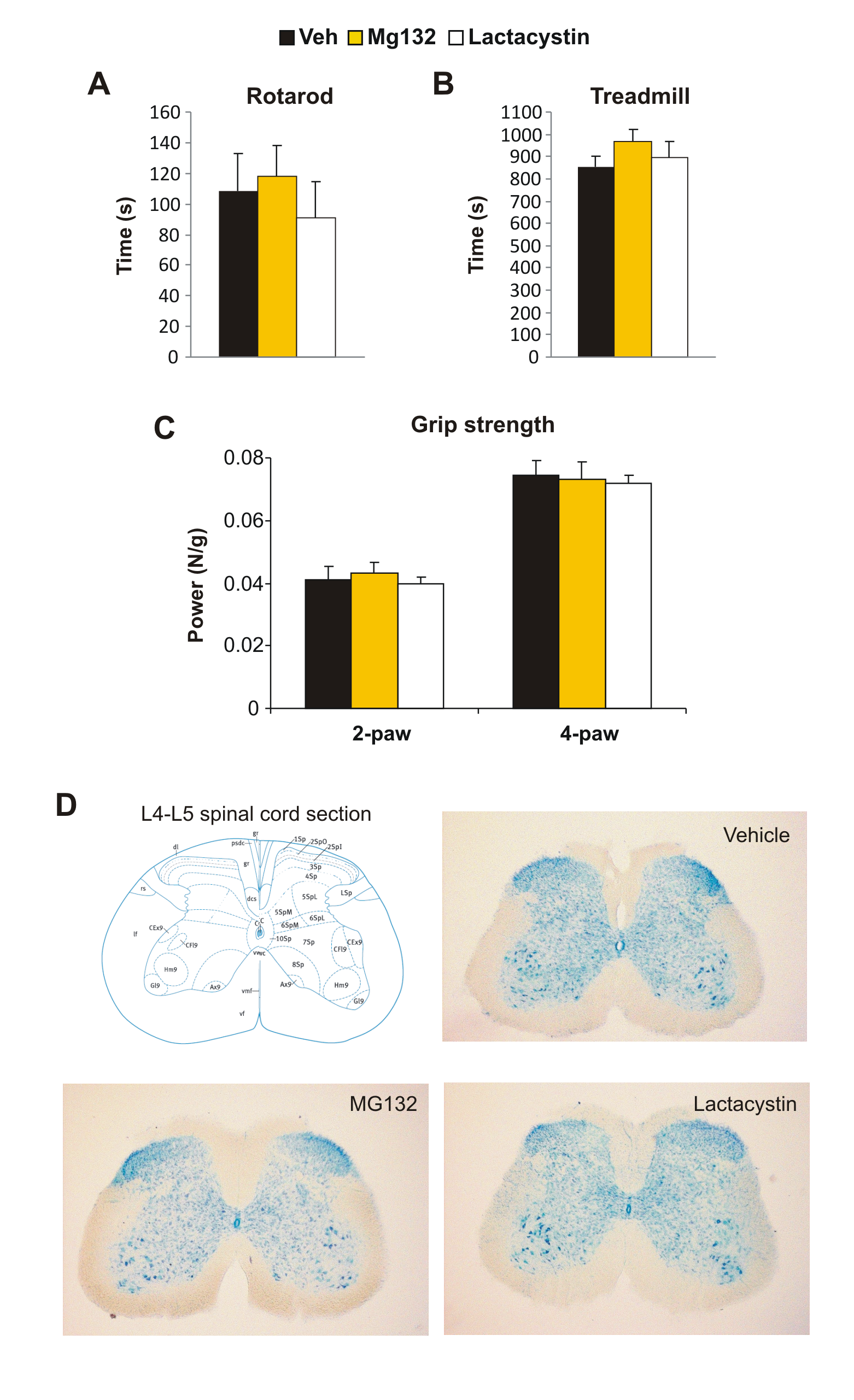

Supplement: Figure S3 — Proteasome inhibition during early postnatal development does not induce motor behaviour or morphological alterations in the spinal cord of adult mice. A–C, Motor function in adult mice that were treated with proteasome inhibitors during early life was measured by their performance in the rotarod (A), treadmill (B) and grip strength (C) tests. D, Representative Nissl staining microphotograph at lumbar 4–5 level of spinal cord is shown (D). (TIF) [file pone.0028927.s003.tif]
