# Supplementary material for: Postnatal Proteasome Inhibition Induces Neurodegeneration and Cognitive Deficiencies in Adult Mice: A New Model of Neurodevelopment Syndrome
Source: PLoS One. 2011 Dec 12;6(12):e28927. doi: 10.1371/journal.pone.0028927 (PMC3236230; doi:10.1371/journal.pone.0028927)
Supplement: Table S1 — Total object exploration times (in seconds) of adult mice injected postnatally with the vehicle alone, MG-132 or lactacystin in each session of a 15-minute OR memory test. STM, short-term memory; LTM, long-term memory. (DOC) [file pone.0028927.s004.doc]

**Table 1. Total object exploration times (in seconds) of adult mice injected postnatally with the vehicle alone, MG-132 or lactacystin in each session of a 15-minute OR memory test. STM, short-term memory; LTM, long-term memory**

|  | **Training** | **STM** | **LTM** |
| --- | --- | --- | --- |
| **Vehicle** | 91.94± 26.74 | 65± 21.55 | 67.15 ± 33.25 |
| **MG132** | 73.33± 15.91 | 44.71 ± 5.58 | 40.14 ± 8.12 |
| **Lactacystin** | 110.9± 34.52 | 93.4 ± 31.87 | 80 ± 16.45 |
| **Statistical analysis** | ns | ns | ns |
