# Supplementary material for: Postnatal Proteasome Inhibition Induces Neurodegeneration and Cognitive Deficiencies in Adult Mice: A New Model of Neurodevelopment Syndrome
Source: PLoS One. 2011 Dec 12;6(12):e28927. doi: 10.1371/journal.pone.0028927 (PMC3236230; doi:10.1371/journal.pone.0028927)
Supplement: Methods S1 — Detailed description of neonatal behavioural tests included in the Fox battery. (DOC) [file pone.0028927.s005.doc]

**Supplementary data**

**Neonatal behavioural tests**

The testing protocols included in the Fox battery (Fox 1965) in which whole litters were separated from the dams and maintained for 30 min in a warm environment. Males and females were pooled for neurodevelopmental screening given that preliminary experiments failed to demonstrate a significant effect of sex on these measures. The range of ages at which responses were observed was evaluated in preliminary experiments in order to define periods of observation and to reduce handling. All testing was performed between 07:30 a.m. and 12:00 a.m. After completing the experimental procedure on each litter, the mice were weighed, injected and returned to the mother.

***Righting reflex***. The animal was placed face up and the time taken to turn over to a prone position with all four feet on the floor was assessed. The maximum time allocated to perform the test was 30 seconds. Scoring was as follows: all four paws were on the floor (3), one or more paws remaining beneath body (2), vigorous but no unsuccessful attempts to right (1), and no response (0).

***Cliff drop aversion***. The mouse was placed on the edge of the table with the forepaws and head extending over the edge. The response was scored as positive if the mouse turned and crawled away at least 45º from the “cliff”. The maximum time allocated to perform this test was 30 seconds.

***Negative geotaxis.*** The animal was placed facing downwards on a 30º incline and the latency to turn 180º was recorded. The maximum time allocated to perform the test was 30 seconds.

***Pivoting activity***. The total number of degrees turned by the pup during a 30 s period was recorded. The test was performed on a flat surface covered with a green paper on which lines had been drawn to delineate four 90º quadrants. The number of degrees was scored only in completed 90º segments.

***Walking test.*** The latency for a mouse to walk a distance exceeding its body length on all four legs was measured on a flat surface covered with green paper.

***Suspension test.*** Mice were suspended by the forelimbs 20 cm above the work surface on a stretched cord about 20cm in length. The time taken to fall was measured up to a maximum of 1 minute.

Reference.

[Fox WM](http://www.ncbi.nlm.nih.gov/pubmed?term="Fox WM"%5BAuthor%5D) (1965) Reflex-ontogeny and behavioural development of the mouse. [Anim Behav](javascript:AL_get(this, 'jour', 'Anim Behav.');) 13: 234-241.
